# Magnetic resonance imaging in the preoperative evaluation of breast cancer patients

**DOI:** 10.1590/0100-3984.2017.50.2e2

**Published:** 2017

**Authors:** Cristine Norwig Galvão

**Affiliations:** 1 MSc, MD, Radiologist at Hospital Unimed Sorocaba and at Clínica IDS de Sorocaba, Sorocaba, SP, Brazil. E-mail: crismagalvao@gmail.com

Despite the undeniable success of mammography in the detection of breast cancer, the method has major limitations, one of which is that it is difficult to detect malignant lesions by mammography, because of the high density of the fibroglandular parenchyma. Another significant limitation is the potential for false-negative results. It has been shown that, in retrospect, approximately 33% of all malignant breast neoplasms eventually detected by mammography could have been identified in previous mammography examinations that were read as negative^(1,2)^.

Since the introduction of magnetic resonance imaging (MRI) in the 1970s, there have been significant advances in techniques for its use in the detection and diagnosis of breast cancer. The first studies involving the use of MRI in the evaluation of breast cancer were conducted in the 1980s. However, those studies dealt with only the intrinsic contrast of tissues in T1- and T2-weighted images, which precluded the diagnosis of the disease. In two independent studies, both published in 1989, Kaiser et al.^(3)^ and Heywang et al.^(4)^ reported that breast tumors previously detected on mammography showed gadolinium-based contrast enhancement on MRI scans, allowing them to be differentiated from background tissue. Another significant MRI finding was the enhancement of tumors that had not been detected on mammography^(1,3,4)^.

Although there is no unified protocol, some basic principles are universally accepted for a good MRI study of the breasts^(1)^: the use of devices with a magnetic field ≥ 1 tesla; the use of dedicated breast coils; and the administration of intravenous contrast with dynamic post-contrast image acquisition.

It is known that MRI has sensitivity above 90% for the detection of invasive breast cancer. However, the benefits of its use in the preoperative staging of breast cancer remain undefined. Despite the fact that MRI is used in daily clinical practice for the detection of breast cancer as well as for the detection of cancer in the contralateral breast, its use does not necessarily improve the clinical outcomes of the patients involved^(5)^. The use of MRI in the preoperative evaluation has resulted in an increase in the number of mastectomies. However, in comparison with patients who do not undergo MRI, those submitted to MRI alone represent a group-comprising young patients and patients with dense breasts, as well as those with genetic mutations, those at high risk, those presenting with tumors that are more aggressive, those undergoing MRI at centers that are more specialized, and those with a high socioeconomic status-in which that radical treatment (mastectomy) is more likely to be used.

As in the international community, numerous scientific studies conducted in Brazil, in the various areas of diagnostic imaging, have made relevant contributions to the study of breast cancer^(6-13)^. There are as yet no consistent data regarding the role of breast MRI in patients with known neoplasia who are eligible for conservative therapy. In view of that, França et al.^(14)^, in an article published in this issue of **Radiologia Brasileira**, attempted to evaluate the role of MRI in treatment planning, comparing MRI, mammography, and ultrasound in terms of the determination of the tumor size, at its greatest diameter, using the actual size of the surgical specimen as the standard. The authors also sought to assess the presence of additional lesions (those not detected in previous examinations) and how such findings could influence the planning of treatment strategies. The actual size of the surgical specimen correlated better with the tumor size determined by MRI than with that determined by the conventional methods. Another aspect was that MRI was able to detect 33.1% of the additional lesions in the same breast or in the contralateral breast, one third of those lesions being malignant, and the treatment strategy was consequently modified in 14.4% of the patients.

The limitations of the França et al.^(14)^ study were that it was a retrospective analysis, that it was not possible to evaluate the tumors in all of the examinations performed by the conventional methods, that there was no standardization of the equipment employed, and that the images were not reviewed. However, the authors stressed that MRI was more accurate in determining the size of the primary tumor at its largest diameter and was efficacious in the detection of additional tumors not visualized on conventional examinations. The discussion section of the article provides an analysis of the current state of this topic, citing several relevant articles, allowing the knowledge in this area to be expanded. The authors highlight the work of Turnbull et al.^(15)^, which was a prospective, randomized, multicenter study aimed at analyzing the clinical efficacy of contrast-enhanced MRI in patients with primary breast cancer. The results obtained to date indicate that the use of MRI alone provides no advantage over the combined use of mammography, ultrasound, and biopsy.
